# GSK3β and VDAC Involvement in ER Stress and Apoptosis Modulation during Orthotopic Liver Transplantation

**DOI:** 10.3390/ijms18030591

**Published:** 2017-03-08

**Authors:** Mohamed Amine Zaouali, Arnau Panisello, Alexandre Lopez, Carlos Castro, Emma Folch, Teresa Carbonell, Anabela Rolo, Carlos Marques Palmeira, Agustin Garcia-Gil, René Adam, Joan Roselló-Catafau

**Affiliations:** 1 Experimental Hepatic Ischemia-Reperfusion Unit, Institut d’Investigacions Biomèdiques de Barcelona (IIBB), Spanish National Research Council (CSIC), Barcelona 08036, Catalonia, Spain; daminzaouali12@yahoo.fr (M.A.Z.); arnau.panisello@iibb.csic.es (A.P.); emma.folch@iibb.csic.es (E.F.); 2 Research Unit of Biology and Molecular Anthropology Applied to Development and Health (UR12ES11), Faculty of Pharmacy, University of Monastir, Monastir 5000, Tunisia; 3 High Institute of Biotechnology of Monastir, University of Monastir, Monastir 5000, Tunisia; 4 Centre Hépato-Biliaire, AP-PH, Hôpital Paul Brousse, Paris 94804, France; alexandregl.lopez@gmail.com (A.L.); ccastrob@gmail.com (C.C.); rene.adam@aphp.fr (R.A.); 5 Department of Physiology, Faculty of Biology, University of Barcelona, Barcelona 08028, Catalonia, Spain; tcarbonell@ub.edu; 6 Center of Neurosciences and Cell Biology, University of Coimbra, Coimbra 3004-504, Portugal; anpiro@ci.uc.pt (A.R.); palmeira@ci.uc.pt (C.M.P.); 7 Hospital Clínico, Zaragoza 50009, Spain; agarciagil@telefonica.net

**Keywords:** IGL-1 preservation solution, trimetazidine, liver transplantation, ischemia–reperfusion injury, GSK3β, VDAC, ER stress

## Abstract

We investigated the involvement of glycogen synthase kinase-3β (GSK3β) and the voltage-dependent anion channel (VDAC) in livers subjected to cold ischemia–reperfusion injury (I/R) associated with orthotopic liver transplantation (OLT). Rat livers were preserved in University of Wisconsin (UW) and Institute Georges Lopez (IGL-1) solution, the latter enriched or not with trimetazidine, and then subjected to OLT. Transaminase (ALT) and HMGB1 protein levels, glutamate dehydrogenase (GLDH), and oxidative stress (MDA) were measured. The AKT protein kinase and its direct substrates, GSK3β and VDAC, as well as caspases 3, 9, and cytochrome C and reticulum endoplasmic stress-related proteins (GRP78, pPERK, ATF4, and CHOP), were determined by Western blot. IGL-1+TMZ significantly reduced liver injury. We also observed a significant phosphorylation of AKT, which in turn induced the phosphorylation and inhibition of GSK3β. In addition, TMZ protected the mitochondria since, in comparison with IGL-1 alone, we found reductions in VDAC phosphorylation, apoptosis, and GLDH release. All these results were correlated with decreased ER stress. Addition of TMZ to IGL-1 solution increased the tolerance of the liver graft to I/R injury through inhibition of GSK3β and VDAC, contributing to ER stress reduction and cell death prevention.

## 1. Introduction

Cold ischemia–reperfusion (I/R) injury is a major cause of primary graft non-function after liver transplantation (LT) [1]. As a preservation solution, the European Liver Transplant Registry, proposed IGL-1 as a good alternative to UW, until now the reference solution in liver transplantation [2]. The composition of the preservation solution is crucial for the viability of liver grafts kept for prolonged ischemic periods [3]. The benefits of IGL-1 solution are mainly associated with the nitric oxide generation and the prevention of endoplasmic reticulum (ER) stress and inflammatory response [4].

In addition, in previous publications, we have shown that the use of additives such as trimetazidine (TMZ) to UW [5,6] and IGL-1 solutions [7,8] contributes to increasing the preservation of steatotic liver grafts (which are more vulnerable than non-steatotic ones) against cold I/R injury [9]. More recently, we proved that the presence of TMZ in IGL-1 solution increases the liver graft protection against cold I/R associated with orthotopic liver transplantation (OLT) by promoting effective prevention of mitochondrial damage [10]. However, the underlying mechanisms of mitochondrial protection responsible for these IGL-1 benefits have not been fully investigated.

Glycogen synthase kinase-3β (GSK3β) is a serine/threonine kinase that participates in the regulation of many cell functions. Its activity is controlled by multiple mechanisms, including its phosphorylation and intracellular translocation. GSK3β is active in its dephosphorylated form, showing a pleiotropic function in the regulation of cell activation, differentiation, and apoptosis [11,12]. In addition, GSK3β is responsible for the phosphorylation of the voltage-dependent anion channel (VDAC), the major resident protein of the outer membrane of the liver mitochondria [13,14,15]. VDAC forms the primary passage for ions and metabolites between mitochondria and cytosol and it is also involved in programmed cell death regulation [13,16].

GSK3β has been implicated in liver I/R pathophysiology. Its inhibition improves liver microcirculation and hepatocellular function after hemorrhagic shock [17]. In addition, its phosphorylation has been implicated in the hepatoprotection induced by ischemic preconditioning against I/R [18]. In a previous study [19], our group described the mitochondrial protection mechanisms involved in this process. More recently, a relationship between GSK3β and ER stress has been evidenced in a pancreatitis model [20]. That study demonstrated that Valproate pretreatment protects pancreatic β-cells from palmitate-induced ER stress and apoptosis by inhibiting GSK3β. Despite the crucial role of GSK3β in a range of pathologies, its involvement in cold I/R associated with liver transplantation remains unclear. Thus, understanding its role in the physiopathology of cold I/R will enhance our knowledge of the basic mechanisms underlying the pathogenesis of this disorder and is likely to aid the identification of new therapeutic avenues.

In this report we assessed the effect of GSK3β modulation by trimetazidine, as an additive in IGL-1 solution, for enhancing liver graft preservation followed by OLT. We also evaluated the relevance of GSK3β inhibition during cold storage for the modulation of ER stress and mitochondrial damage.

## 2. Results

**Figure 1:**
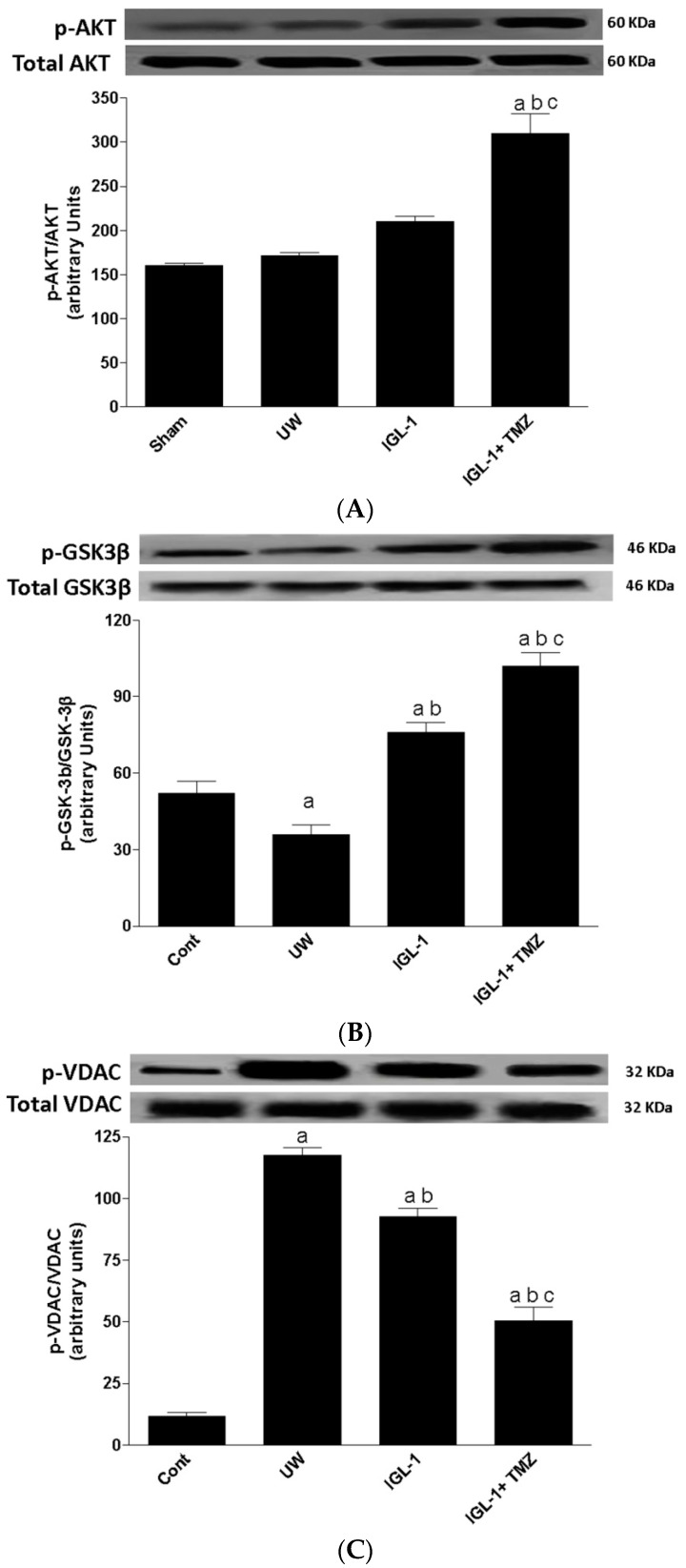
AKT, GSK3β, and VDAC protein levels after transplantation. Representative Western blot at the top and densitometric analysis at the bottom of total and phospho-AKT (**A**), total and phospho-GSK3β (**B**) and phospho-VDAC (**C**). UW: liver preserved in UW solution; IGL-1: liver preserved in IGL-1 solution; IGL-1+TMZ: liver preserved in IGL-1 solution with trimetazidine. ^a^ *p* < 0.05 vs. Sham, ^b^ *p* < 0.05 vs. UW, and ^c^ *p* < 0.05 vs. IGL-1.

In this study we evaluated whether the addition of trimetazidine to IGL-1 solution protected liver grafts against cold I/R associated with liver transplantation, and whether this protection might be mediated by changes in the protein levels of AKT and its direct substrate GSK3β after 8 h of ischemic cold storage followed by 24 h orthotopic liver transplantation. As indicated in Figure 1A, phosphorylated AKT (p-AKT) levels were similar in sham livers and in livers preserved with UW. This was associated with a decrease in the protein levels of phosphorylated GSK3β (p-GSK3β), the inactive form of GSK3β, in livers preserved in UW solution (Figure 1B). IGL-1 solution increased p-GSK3β compared with UW; however, addition of TMZ to IGL-1 solution increased p-AKT protein levels, which were correlated with reduced GSK3β activity by increasing its phosphorylation (Figure 1A,B). Total AKT and GSK3β were unchanged in all groups.

In view of the potential relationship between GSK3β and VDAC, one of the major resident proteins of the outer membrane mitochondria (the active non-phosphorylated GSK3β activates VDAC by increasing its phosphorylation) [21], we next evaluated whether changes in GSK3β activity might be associated with changes in mitochondrial VDAC activity. In fact we observed that phosphorylated VDAC (p-VDAC) pattern profiles were the reverse of those obtained in p-GSK3β. As indicated in Figure 1C we noted an increase in p-VDAC protein levels in livers preserved in UW preservation solution when compared to sham. The addition of TMZ to IGL-1 reduced VDAC phosphorylation when compared to UW solution and IGL-1 solution alone.

These findings were consistent with the reductions in mitochondrial damage, oxidative stress and liver injury evidenced respectively by reductions in GLDH activity, MDA, and ALT (Table 1).

**Figure 2:**
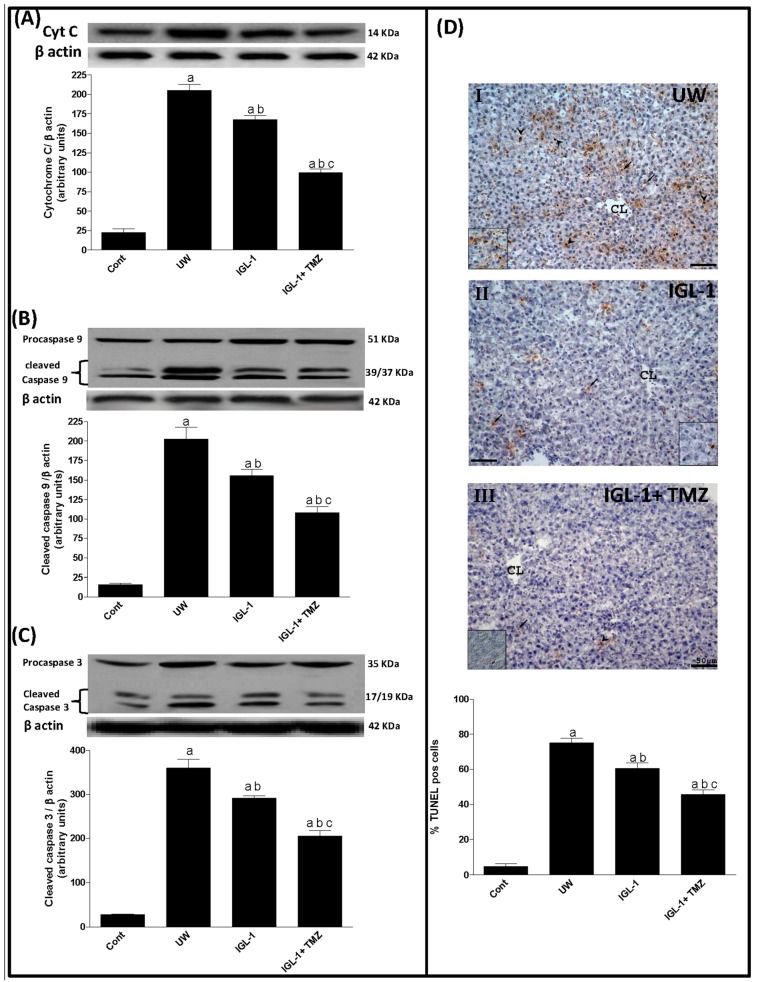
Cell death after liver transplantation. Representative Western blot at the top and densitometric analysis at the bottom of protein levels of cytochrome C (**A**), cleaved caspase 9 (**B**), and cleaved caspase 3 (**C**) expression in liver grafts; and (**D**) Representative light photomicrographs of Terminal deoxynucleotidyl transferase dUTP nick end labeling (TUNEL) stained sections. Livers submitted to Orthotopic liver transplantation (OLT) after cold storage preservation with UW (**I**) showed numerous positive cells, both hepatocytes (arrows) and sinusoidal lining cells (arrow heads). The positivity decreased when the livers were submitted to OLT after cold storage preservation with IGL-1 solution (**II**); The addition of TMZ to IGL-1 solution (**III**) induced a further reduction of TUNEL positivity both for hepatocytes and sinusoidal lining cells. UW: liver preserved in UW solution; IGL-1: liver preserved in IGL-1 solution; IGL-1+TMZ: liver preserved in IGL-1 solution with trimetazidine. ^a^ *p* < 0.05 vs. Sham; ^b^ *p* < 0.05 vs. UW; and ^c^ *p* < 0.05 vs. IGL-1. CL: centrolobular vein. Scale bar (black) indicates 50 μm.

Since the mitochondrial alteration was generally correlated with apoptotic cell death after liver transplantation, we evaluated the changes in protein levels of cytochrome C and cleaved caspase 3 and 9 as well as the percentage of TUNEL-positive cells. Our results indicated that the activation of GSK3β and VDAC in UW group was in accordance with the increased protein levels of cytochrome C and cleaved caspase 3 and 9 compared with the sham group (Figure 2A–C). Although the release of cytochrome C was prevented more in IGL-1 than UW, the lowest levels were observed when TMZ was added to IGL-1 (Figure 2A). This was also confirmed by the fall in the percentage of TUNEL-positive cells as well as in cleaved caspases 3 and 9 in the IGL-1+TMZ group compared with UW and IGL-1 groups (Figure 2B–D). These results suggest that the inactivation of GSK3β phosphorylation could modulate VDAC activity, thus reducing cytochrome C release and caspase 3 and 9 cleavage.

**Figure 3:**
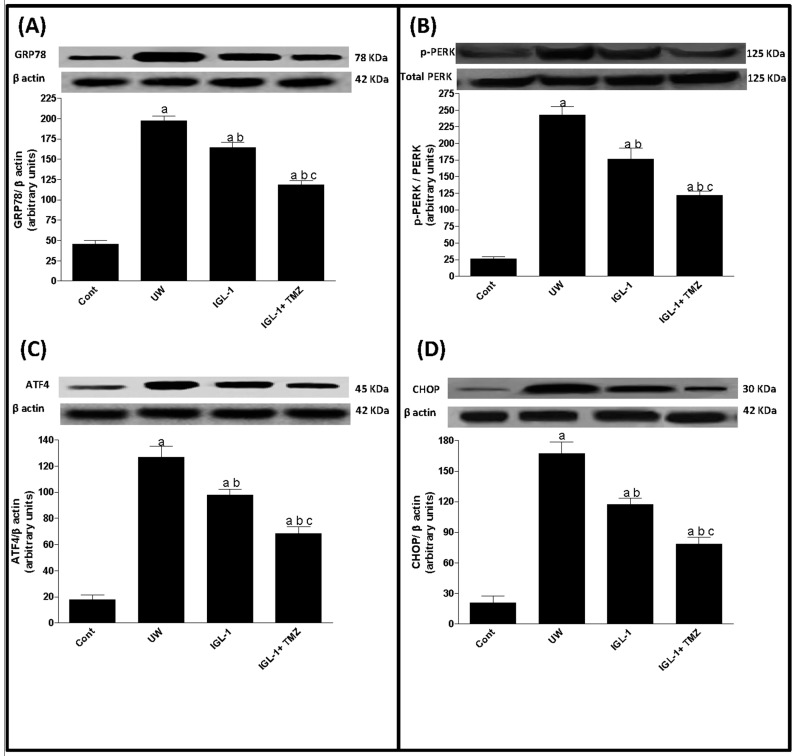
Endoplasmic reticulum stress after liver transplantation. Representative Western blot at the top and densitometric analysis at the bottom of (**A**) GRP78, (**B**) total and phospho-PERK, (**C**) ATF4 and (**D**) Chop. UW: liver preserved in UW solution; IGL-1: liver preserved in IGL-1 solution; IGL-1+TMZ: liver preserved in IGL-1 solution with trimetazidine. ^a^ *p* < 0.05 vs. Sham; ^b^ *p* < 0.05 vs. UW; and ^c^ *p* < 0.05 vs. IGL-1.

There is growing evidence that perturbations in the endoplasmic reticulum (ER) are novel subcellular effectors, possibly involved in the promotion of cell death during cold organ preservation [22]. For this reason we evaluated the effects of the different solutions on ER stress. We assessed hepatic protein levels of GRP78, phospho-PERK (p-PERK), ATF4, and CHOP by Western blotting. Our results demonstrated increased levels of GRP 78 (Figure 3A) and p-PERK (Figure 3B) concomitantly with ATF4 and CHOP accumulation (Figure 3C,D) when UW solution was used. However, the activation of GRP78, p-PERK, ATF4, and CHOP was significantly lower in the IGL-1 solution and was prevented even further by the addition of TMZ to IGL-1 solution (Figure 3A–D).

**Figure 4:**
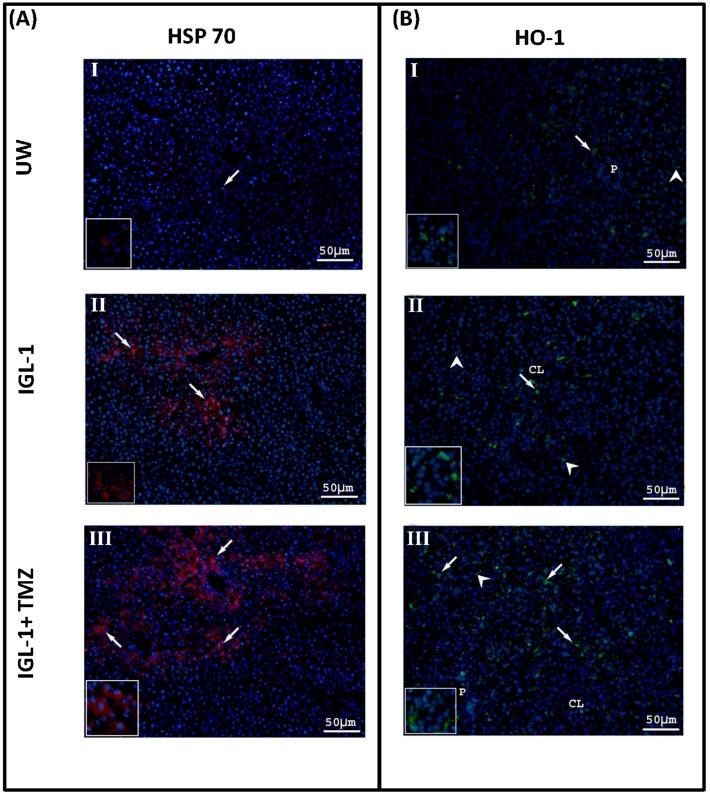
Representative light photomicrographs of HSP70 (**A**) and HO-1 (**B**) immunohistochemistry. For HSP70 (**A**) the positivity occurred in parenchymal cells, while non-parenchymal cells displayed practically no staining, and were mainly distributed in the periportal areas. Only a few scattered hepatocytes were positive to HSP70 staining in livers submitted to OLT after cold storage preservation with UW (**I**); the positivity increased when the livers were submitted to OLT after cold storage with IGL-1 solution (**II**), and even more when trimetazidine (IGL-1+TMZ) was added to IGL-1 solution (**III**). Concerning HO-1, (**B**) the positivity occurred both in parenchymal cells (arrows) and in sinusoidal lining cells (arrow heads) and it was homogeneously distributed throughout the lobule. Livers submitted to OLT after cold storage preservation with UW (**I**) showed only scarce positivity. The positivity increased when the livers were submitted to OLT after cold storage preservation with IGL-1 solution (**II**). The addition of TMZ to IGL-1 solution (IGL1+TMZ) (**III**) induced a further increase of HO-1 expression both in hepatocytes and in sinusoidal lining cells. CL: centrolobular vein; P: portal vein. Scale bar 50 μm.

The benefits of ER stress reduction achieved by using TMZ as an additive to IGL-1 were correlated with an increased expression of other cytoprotective factors such as HO-1 and HSP70. As shown in Figure 4, the increases in HO-1 and HSP70 protein levels occurred in livers preserved in IGL-1 enriched with TMZ were larger than those achieved with UW or IGL-1 alone.

**Figure 5:**
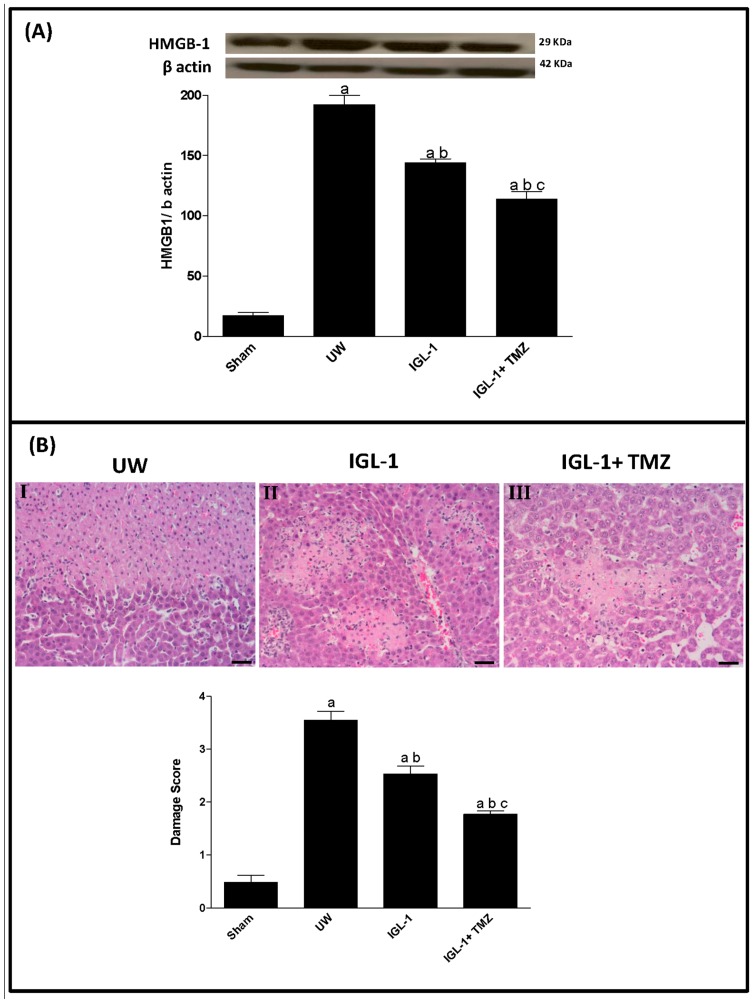
Inflammatory response and histological lesions in the liver. (**A**) Representative Western blot at the top and densitometric analysis at the bottom of HMBG1 protein levels; (**B**) Histological damage after liver transplantation from grafts preserved in UW (**I**), IGL-1 (**II**) and IGL-1+TMZ (**III**) preservation solutions. (**I**) Liver preserved in UW solution: extensive and confluent areas of coagulative hepatic necrosis with neutrophil infiltration; (**II**) Liver preserved in IGL-1 solution: reduced areas of coagulative hepatic necrosis with neutrophil infiltration; and (**III**) Liver preserved in IGL-1+IGL-1 solution: TMZ induced further reductions in areas of coagulative hepatic necrosis with neutrophil infiltration. UW: liver preserved in UW solution; IGL-1: liver preserved in IGL-1 solution; IGL-1+TMZ: liver preserved in IGL-1 solution with trimetazidine. Scale bar (Black) indicates 50 μm. ^a^ *p* < 0.05 vs. sham; ^b^ *p* < 0.05 vs. UW; and ^c^ *p* < 0.05 vs. IGL-1.

Finally, because of the association of inflammatory response and hepatocellular damage in liver transplantation with ER stress response, we explored the expression of HMGB-1, a well-known early mediator in inflammation and liver damage after liver transplantation [23], in a histological study. In fact we noted increased HMGB-1 protein levels in livers preserved in UW solution compared with sham. Although the use of IGL-1 solution kept HMGB-1 protein levels down compared with UW solution, the enrichment of IGL-1 with TMZ reduced HMGB-1 accumulation even further (Figure 5A). This was in agreement with the histological results (Figure 5B) which demonstrated a pattern similar to that described for HMGB-1 protein levels.

## 3. Discussion

It is well established today that cold I/R injury is a major cause of primary graft non-function after liver transplantation [1]. The composition of preservation solution is crucial for the viability of liver grafts kept for prolonged ischemic periods and then subjected to transplantation [2]. The role of GSK3β in the pathophysiology of liver I/R injury has not been investigated thoroughly. GSK3β is a serine/threonine kinase whose activity is inhibited by phosphorylation [12]; recently, it was demonstrated that its inhibition improves liver microcirculation and hepatocellular function after hemorrhagic shock [17]. A phosphorylated GSK3β increase is also involved in the adaptive mechanisms of hepato-protection against I/R injury induced by ischemic preconditioning in the liver [18]. Moreover, it has been reported that modulation of Akt and its downstream targets GSK3β and VDAC could be used as a potential therapeutic strategy for protecting the kidney against I/R injury [24].

In view of the recent data published by the European Liver Transplant Registry [2] and our previous reports of liver preservation solutions [25], we evaluated the relevance of the AKT/GSK3β pathway and its direct substrate VDAC in rat orthotopic liver transplantation settings using UW, IGL-1 and IGL-1+TMZ preservation solutions. Our data demonstrated, for the first time, the relevance of GSK3β phosphorylation in liver transplantation and how its activity also depends on the preservation solution used. In fact, the inactivation of GSK3β phosphorylation is a feature of the protection provided by IGL-1, but not of the protection provided by to UW. The differences in phosphorylated GSK3β could be explained by the increased p-AKT protein levels especially when TMZ was added to IGL-1. The regulation of GSK3β by AKT is well documented [21,26]. In fact previous studies suggested that protein kinase C and Akt are mainly responsible for GSK-3β phosphorylation in ischemic preconditioning-induced cardioprotection [21,26]. Along the same lines, several reports have associated the protective effect of TMZ against I/R injury with its ability to activate AKT, which represents a major pathway mediating cell survival [27,28,29].

In addition, it is well established that phosphorylation of mitochondrial VDAC is a key element of the complex network of regulatory and signaling pathways involved in mechanism for numerous cellular processes, such as those occurring in the mitochondrial dysfunction associated with liver transplantation [16,30]. For this reason we assessed the impact of GSK3β phosphorylation on VDAC modulation. Our results show that TMZ addition to IGL-1 solution protected mitochondria from the deleterious consequences of cold I/R injury via promotion of GSK3β phosphorylation and prevention of VDAC phosphorylation. These results are in accordance with previous studies which demonstrated that inhibition of GSK3β phosphorylation was associated with a decrease in VDAC phosphorylation and delayed mitochondrial permeability transition pore (mPTP) opening after I/R injury [21,30]. Furthermore, TMZ acts on mitochondria by restoring ATP synthesis and by maintaining the impermeability of the mitochondrial membrane [31]. Indeed, TMZ might interact with VDAC which, it has also been suggested, may control the release of cytochrome C [32]. Taken this into account, it could be argued that the inactivation of phosphorylation of GSK3β by TMZ during cold ischemia may facilitate hypoxic adaptation through the phosphorylation of VDAC.

Given the close relationship between mPTP opening and apoptotic cell death, we next evaluated the key mediators of mitochondrial-induced apoptosis such as cytochrome c, cleaved caspase 3 and 9 levels and the percentage of TUNEL-positive cells. Our results indicated that IGL-1 prevented cytochrome C release and consequently reduced caspase 3 and 9 activation when compared with UW solution. IGL-1 enrichment with TMZ enhanced mitochondrial protection even more and inhibited apoptosis. These results are in agreement with other data suggesting that TMZ exerts its cytoprotective effect during early reperfusion essentially by reducing the mitochondrial dysfunction after renal I/R [33].

More recently, ER stress alteration has been proposed as a new contributor to the poor tolerance of livers to I/R injury [22,34]. It has been reported that ER stress is a mediator of post-transplant injury in liver grafts, thus promoting cell death [4]. Here we demonstrate for the first time that the addition of TMZ to IGL-1 preservation solution reduces ER stress in liver grafts, thus enhancing liver function after transplantation. In fact the impact of TMZ on ER stress modulation in liver graft remains unclear. Indeed, data from another study suggest that ER stress may not be a target through which TMZ exerts its cytoprotective effect against warm I/R in kidney [33]. The reasons for the discrepancies in the findings of the two studies might be in part related to the differences in the organs and experimental protocols used, including the type of ischemic injury (warm or cold). Although the effect of TMZ on ER stress modulation was not fully explored in this study, we suggest that TMZ may reduce ER stress via GSK3β inhibition. In fact, it is well known that ER depends on ATP to correct the misfolding of protein errors; ATP reduction following I/R injury can lead to ER stress and cell death [35]. Thus the enhancement of mitochondrial protection by the use of TMZ could reduce ER stress. This finding may be supported by the demonstration of a relation between GSK3β and ER stress in a pancreatitis model [20]. This study showed that Valproate pretreatment protects pancreatic β-cells from palmitate-induced ER stress and apoptosis by inhibiting glycogen synthase kinase-3β [20]. Similarly, another study demonstrated that Akt activation attenuated lipopolysaccharide-induced cardiac dysfunction via Akt/GSK3β-dependent inhibition of apoptosis and ER stress [36].

Finally, in order to assess the impact of AKT/GSK3β pathway modulation on inflammation and liver damage after orthotopic liver transplantation we examined changes in the protein levels of HMGB-1, an early inflammatory mediator after acute I/R injury [23,37], as well as the structural alteration (histological study). We found that the addition of TMZ to IGL-1 reduced HMGB-1 protein levels compared with UW and IGL-1 alone, and that this reduction was correlated with reduced structural damage. Our results corroborate those of previous studies suggesting that inflammatory signaling following hepatic I/R is triggered by the release of the nuclear protein HMGB-1from necrotic or damaged cells, which acts as a proinflammatory cytokine and functions as a major stimulus of necrosis-induced inflammation [23,38].

In summary, IGL-1 solution, especially when supplemented with TMZ, increases the tolerance of the liver graft to I/R associated with OLT through the inhibition of GSK3β and VDAC, thus preventing cell apoptosis. GSK3β inhibition contributes to reinforcing mitochondrial protection and leads to reduced ER stress. The inhibition of GSK3β is an issue of growing interest in the search for new therapeutic strategies for the prevention of I/R injury.

## 4. Materials and Methods

### 4.1. Experimental Animals

Liver transplantation protocol was applied to male Sprague–Dawley rats (250–300 g bodyweight) under general anesthesia with isofluorane respecting the European Union rules for animal experiments (EC-guideline 86/609/CEE) and approved by the Ethics Committees for Animal Experimentation (CEEA)of the University of Barcelona (number 483/16).

### 4.2. Experimental Design

The following experimental groups were defined as follows.

Group 1 (Sham: *n* = 12): Sprague–Dawley rats were immediately subjected to laparotomy and the subsequent ligation of suprarenal and diaphragmatic vein and hepatic artery, respectively. Group 2 (UW: *n* = 12, six transplantation): donor livers were flushed and stored in UW preservation solution for 8 h at 4 °C, and then underwent OLT according to Kamada’s cuff technique without hepatic artery construction [39,40]. Rats were sacrificed 24 h after reperfusion for liver and plasma sample collection. Group 3 (IGL-1; *n* = 12, six transplantation): same as 2, but using IGL-1 preservation solution instead of UW. Group 4 (IGL-1+TMZ: *n* = 12, six transplantation): same as 3, but IGL-1 was supplemented with TMZ at 10^−6^ mol/L [7].

### 4.3. Biochemical Determinations

#### 4.3.1. Transaminase Assay

Hepatic injury was evaluated by alanine aminotransferase (ALT) levels using commercial kits from RAL (Barcelona, Spain). ALT was determined at 365 nm with an Ultraviolet spectrophotometer (Beckman Coulter, Inc., Fullerton, CA, USA) and calculated following the supplier’s instructions [41].

#### 4.3.2. Glutamate Dehydrogenase Activity

Activity of glutamate dehydrogenase (GLDH), a mitochondrial enzyme, was used as an indirect determination of mitochondrial damage, as previously reported [41,42].

#### 4.3.3. Lipid Peroxidation

Liver lipoperoxides were determined by measuring the formation of malondialdehyde (MDA) with the thiobarbiturate reaction, as previously described [42].

#### 4.3.4. TUNEL Assay

The evaluation of DNA fragmentation was carried out by using TUNEL assay, as previously indicated in our studies [4,8].

#### 4.3.5. Histology

The severity of hepatic injury was assessed in the haematoxylin and eosin-stained sections by a point-counting method using optical microscopy. Liver injury was scored on an ordinal scale as previously reported [34].

#### 4.3.6. Western Blotting Analysis

Liver tissue was homogenized as previously described [4,34]: 50 μg proteins were separated on 6%–15% sodium dodecyl sulfate polyacrylamide gel electrophoresis (SDS-PAGE) gels. Proteins were blotted into polyvinylidene fluoride (PVDF) membranes (Biorad, Madrid, Spain) and immunoblotted overnight at 4 °C with antibodies directed against total and phospho-AKT (Ser473), cleaved caspase 3, cleaved caspase 9, total and phospho-GSK3β (ser 9), cytochrome C, and phospho-VDAC (Cell Signaling, Beverly, MA, USA); GRP78, CHOP, ATF4 and total and phospho-PERK (Santa Cruz Biotechnology, Santa Cruz, California, USA), HMGB-1(Abcam, Milton, Cambridge, UK), and β actin (Sigma Chemical, St. Louis, MO, USA). After washing, membranes were incubated with appropriate peroxidase-conjugated secondary antibody for 1 h. Immuno-labeled bands were detected using Western-Bright ECL-HRP Substrate (Advansta, Menlo Park, CA, USA) and quantified using the Quantity One software for image analysis. Results were expressed as densitometry ratios between the protein of interest and the loading control (β-actin).

#### 4.3.7. Heme-Oxygenase (HO-1) Immunohistochemistry

Frozen liver sections (16 μm thick) were fixed in pre-cooled acetone for 10 min at room temperature. After washing and permeabilization, HO-1 was detected by using a primary HO-1 antibody (Sigma Chemical). Following, rinsing in Phosphate-buffered saline solution (PBS) permeabilization was performed again three times with 0.5% Triton X-100 for 10 min. The secondary antibody (Alexa 488 Fluor labeled, donkey anti-rabbit, Invitrogen, Waltham, MA, USA) was applied at the dilution of 1:400 in the blocking solution for 50 min at room temperature. Negative controls were prepared as previously described [8] and images were detected using Nikon Eclipse E1000 fluorescence microscope (Nikon Instruments Inc., Melville, NY, USA).

#### 4.3.8. Heat Shock Protein (HSP-70) Immunohistochemistry

Frozen liver sections (16 μm thick) were fixed in 1% p-formaldehyde for 10 min at room temperature. After washing with PBS, post-fixation with ethanol:acetic acid 2:1 for 5 min and permeabilization twice with 0.5% Triton X-100 for 15 min were performed. Antibody especific binding was blocked for 1 h in PBS with 10% normal goat serum, 3% bovine serum albumin, 1.5% NaCl and 0.5% Triton X-100 for 1 h. Immunohistochemistry analyses were performed using HSP70 (primary anti-body from BD Science, San Jose, CA, USA) and Alexa 594 Fluor labelled (secondary antibody from Invitrogen, USA), as previously described [8]. Negative controls were prepared by replacing the first antibody with phosphate saline buffer. Images were obtained with a Nikon Eclipse E1000 fluorescence microscope [8].

### 4.4. Statistics

Data are expressed as means ± standard error, and were compared statistically by variance analysis, followed by the Student–Newman–Keuls test (Graph Pad Prism software, Inc., La Jolla, CA, USA). *p* < 0.05 was considered significant.

## Figures and Tables

**Table 1:** Serum levels of ALT (Liver injury) and GLDH (mitochondrial damage) and lipid peroxidation (MDA) in liver grafts preserved in UW, IGL-1 and IGL-1+TMZ solutions and then subjected to orthotopic liver transplantation. ^a^ *p* < 0.05 vs. Sham; ^b^ *p* < 0.05 vs. UW; and ^c^ *p* < 0.05 vs. IGL-1.

Parameters	Sham	UW	IGL-1	IGL-1+TMZ
ALT (U/I)	56.12 ± 34.25	565.24 ± 63.73 ^a^	278.87 ± 61.37 ^a,b^	164.14 ± 18.84 ^a,b,c^
GLDH (U/I)	9.13 ± 2.45	131.72 ± 15.26 ^a^	92.21 ± 11.75 ^a,b^	42.78 ± 7.97 ^a,b,c^
MDA (nmol/mg prot)	0.4 ± 0.16	2.55 ± 0.55 ^a^	1.87 ± 0.33 ^a,b^	1.2 ± 0.29 ^a,b,c^
